# From Carbon-Monoxide Inhibition to Light Activation: Probing [NiFe] Hydrogenase Dynamics by Multiscale Time-Resolved Infrared Spectroscopy

**DOI:** 10.1021/acs.inorgchem.6c01321

**Published:** 2026-06-18

**Authors:** Malin Khalil, Elizaveta Kobeleva, Cornelius C. M. Bernitzky, Partha Malakar, Sayantan Bhattacharya, Sagie Katz, Hiroaki Matsuura, Ingo Zebger, Gregory M. Greetham, Hideaki Ogata, James A. Birrell, Marius Horch

**Affiliations:** † Department of Physics, 9166 Freie Universität Berlin, Ultrafast Dynamics in Catalysis, Arnimallee 14, 14195 Berlin, Germany; ‡ STFC Central Laser Facility, 97008 Research Complex at Harwell, Rutherford Appleton Laboratory, Harwell Campus, Didcot, OX11 0QX, U.K.; § Department of Chemistry, 26524 Technische Universität Berlin, Spectroscopic Characterization of Metalloproteins, Straße des 17. Juni 135, 10623 Berlin, Germany; ∥ Graduate School of Life Science, 12744 University of Hyogo, Koto 3-2-1, Kamigori, Ako 678-1297, Hyogo, Japan; ⊥ School of Life Sciences, 98451 University of Essex, Wivenhoe Park, Colchester CO4 3SQ, U.K.

## Abstract

Hydrogenases are metalloenzymes that catalyze the reversible splitting of dihydrogen (H_2_), a clean and sustainable fuel. In this study, we investigate the reversible photodissociation and rebinding of an extrinsic carbon monoxide (CO) ligand at the active site of a [NiFe] model hydrogenase. CO acts as a catalytic inhibitor of the enzyme, whereas its photolysis restores an active state capable of H_2_ binding. Using UV_pump_-IR_probe_ spectroscopy in a multiple-probe configuration that allows covering picosecond to millisecond time scales, we characterize the reaction dynamics following CO photolysis. The results reveal a large temporal window between rapid CO dissociation and slow rebinding, enabling the detailed investigation of H_2_ binding and activation at the active site, unaffected by H_2_ mass transport limitation.

## Graphical abstract

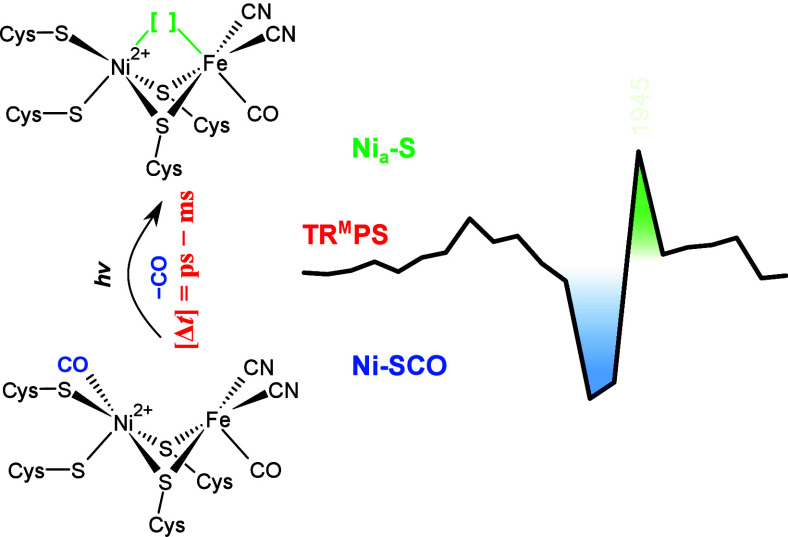

In the search for clean and sustainable energy, one powerful example lies in hydrogenases, metalloenzymes used by microorganisms to catalyze the reversible cleavage of dihydrogen (H_2_).^1^ There are three major classes of hydrogenases distinguished by their active-site metal content: [NiFe], [FeFe] and [Fe] hydrogenases.^2^ The catalytic center of [NiFe] hydrogenase consists of Ni and Fe ions coordinated by four cysteine thiolates.^3,4^ Two cysteines bridge the metal ions, and the other two coordinate solely to Ni. In addition, two CN^–^ groups and one CO are coordinated to Fe.^5^ In certain redox states, the enzyme accommodates an additional bridging ligand between the two metal ions.^1,4^ Infrared (IR) spectroscopy has been used as a powerful tool to study redox-structural changes in hydrogenases,^6–9^ since CN^–^ and CO ligands can be utilized as site-specific, spectrally isolated and structurally sensitive IR probes. This approach has also been used in time-resolved experiments,^10–16^ but studying the catalytic mechanism of these enzymes with high time resolution remains challenging, especially for H_2_ activation.

In a time-resolved ensemble experiment, synchronization is required to start the reaction for all studied molecules at the same time in order to obtain reasonable populations of the formed intermediates. While it is comparably easy to achieve this with short laser pulses for photo processes, rapidly triggering ground-state reactions is hard to achieve. However, hydrogenases are light-sensitive in multiple active and inactive states,^17–22^ which enables their rapid and reversible transformation by light into other catalytic intermediates. This allows studying individual elementary steps along the catalytic cycle with high time resolution. Studying the entire catalytic cycle poses another challenge associated with mass transport of H_2_. H_2_ diffuses toward the active site through a narrow hydrophobic gas channel. If this process is slow relative to the first catalytic steps of H_2_ binding and cleavage, the associated intermediates will not be populated significantly, so that these steps cannot be studied. Notably, this problem is of an inherent kinetic nature and cannot be solved by increasing the time-resolution of the spectroscopic apparatus used. It can be circumvented, though, by adequate design of the time-resolved experiment. The [NiFe] hydrogenase from *Desulfovibrio vulgaris* Miyazaki F (*Dv*MF) is inhibited by carbon monoxide (CO), forming an adduct known as Ni-SCO (Figure 1, left).^23^ While the intrinsic Fe-bound CO ligand of [NiFe] hydrogenase is photostable under all tested conditions, previous studies at cryogenic temperatures have demonstrated that the extrinsic Ni-bound CO ligand can be photolyzed from the Ni-SCO state, resulting in the formation of the catalytic Ni_a_-S intermediate (Figure 1, right).^24^ Thus, by rapidly photolyzing CO, i.e. activating the enzyme, with ultrashort pulses of light in the presence of H_2_, both challenges could be circumvented if the intramolecular gas channel is saturated with H_2_ when the reaction is started.

**Figure 1:**
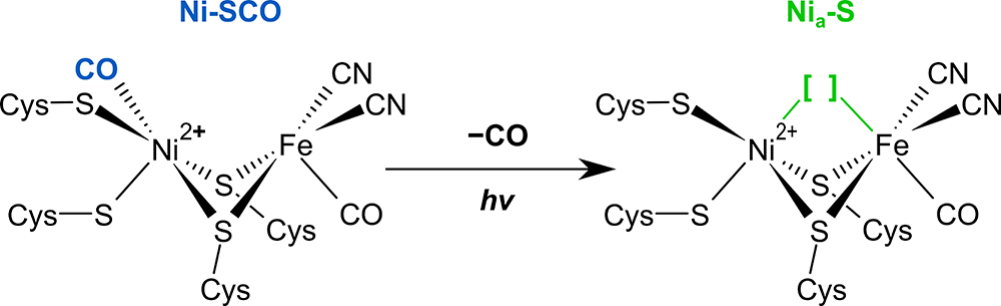
Schematic representation of Ni-SCO and Ni_a_-S states.

Realization of this approach requires that the Ni-SCO state can be (1) enriched and (2) stabilized in the presence of H_2_. Both can be achieved for the [NiFe] hydrogenase from *Dv*MF (Figures S1 and S2), which makes this enzyme an ideal model system for pursuing this strategy. In addition, the time scales of CO photolysis and rebinding must be compatible with those of H_2_ binding and activation at ambient temperature. So far, these time scales are unknown.

In the present work, we apply time-resolved UV_pump_-IR_probe_ spectroscopy in a multiple-probe scheme that allows covering subpicosecond to millisecond time scales.^25–27^ In this method, an UV laser pulse electronically excites the sample, and a synchronized IR pulse train monitors the subsequent dynamics. This approach allows studying – in real time and at ambient temperature – the rate of CO photolysis from the active site and its subsequent rebinding.

The sample was electronically excited using a 250 fs UV pump pulse (350 nm), and IR spectra were recorded under two conditions: (1) with the sample excited by the pump pulse (pump-on spectra) and (2) without excitation (pump-off spectra). To study how the system evolves from the electronically excited state, difference spectra were calculated that reflect the absorbance change of the sample. Here, negative signals reflect the bleaching of the overall ground state of the system in the initial Ni-SCO state, while positive features reflect all species formed via photophysical or photochemical processes after electronic excitation. All signals discussed in the manuscript reflect the stretching vibration of the intrinsic Fe-bound CO ligand, which is the most intense and structurally sensitive reporter for the state of the [NiFe] site. While the Ni-SCO state can also be identified by the stretching vibration of the extrinsic Ni-bound CO ligand (Figures S1 and S2), the high frequency and low intensity of the associated fundamental transition render this mode less suitable for quantitatively tracking the Ni-SCO population. Therefore, the time evolution of all state populations is obtained from the intensity of the stretch mode of the intrinsic Fe-bound CO ligand. The reported time-resolved measurements cover time scales ranging from picoseconds to milliseconds, with different processes dominating at each regime. For clarity, these time scales will be discussed separately.

Figure 2A shows a two-dimensional plot of absorbance change vs delay time (0–500 ps) for the [NiFe] hydrogenase from *Dv*MF enriched in the Ni-SCO state. Spectra recorded at selected time delays are shown in Figure 2B. The negative signal at 1942 cm^–1^ is attributed to ground-state bleaching (GSB) of the Ni-SCO state, consistent with the linear IR absorption spectrum (Figure S1) and the characteristic CO stretching fundamental vibration of the Ni-SCO state reported in the literature.^24^ The positive signal at 1915 cm^–1^, shifted by – 27 cm^–1^ from the GSB peak, is assigned to excited-state absorption (ESA) from the first to the second vibrationally excited state (1–2) of the CO stretching vibration. The frequency shift agrees well with the anharmonicity values reported for the CO stretching vibration in previous IR_pump_–IR_probe_ and two-dimensional infrared spectroscopic studies on other [NiFe] and [FeFe] hydrogenases as well as further monocarbonyl compounds.^28–34^ Similarly, the positive peak at 1890 cm^–1^, additionally shifted by −25 cm^–1^, is assigned to excited-state absorption from the second to the third excited state (2–3) of the CO stretching vibration. The nearly constant spacing of the different transition fits to the behavior of a Morse oscillator, as expected for a bond localized CO stretch vibration.^29^ The positive peak at 1945 cm^–1^ corresponds to the Ni_a_-S state that is formed after CO photolysis.^24^ Of note, this process occurs rapidly on picosecond time scales, as detailed below, indicating that the enzyme is rapidly activated in a photochemical manner.

**Figure 2:**
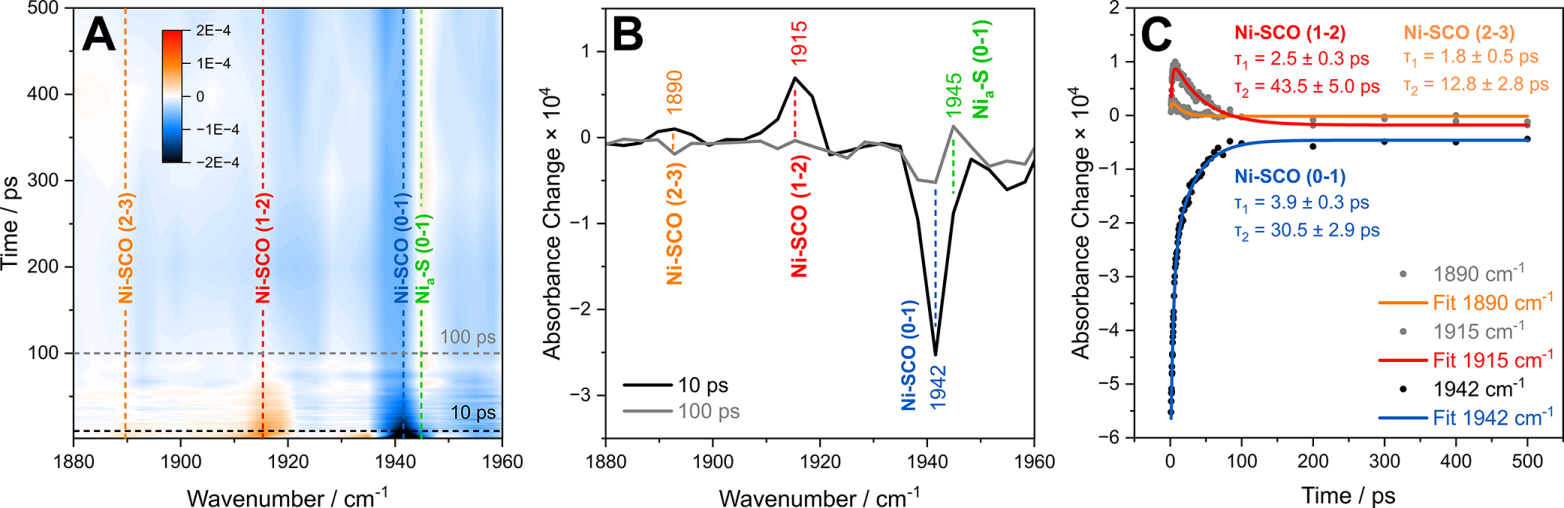
UV_pump_-IR_probe_ data of the [NiFe] hydrogenase from *Dv*MF, enriched in the Ni-SCO state, recorded over picosecond time scales after 350 nm excitation. (A) Contour plot of all time-resolved spectra. (B) Selected spectra obtained at *t* = 10 ps and *t* = 100 ps, respectively. (C) Time-evolution of signal intensities obtained at the indicated frequencies and corresponding fits. For the ground-state bleach signal, both time constants reflect decay components, while for excited-state absorption signals, the shorter time constants reflect a rise of the signals.

From kinetic analysis of the GSB peak, we notice that it does not recover fully on the picosecond time scale (Figure 2C). The incomplete recovery was well fitted using a biexponential function with recovery time constants of τ_1_ = 3.9 ps and τ_2_ = 30.5 ps. Kinetic analysis of the ESA (1–2) signal was performed by fitting a consecutive first-order reaction model to the data.^35^ The fitting yields time constants of τ_1_ = 2.5 ps (rise) and τ_2_ = 43.5 ps (decay). In a similar way, the ESA (2–3) signal was analyzed, yielding τ_1_ = 1.8 ps (rise) and τ_2_ = 12.8 ps (decay). In order to avoid contamination of the kinetics by the coherent time zero artifact, kinetic traces start from the maximum of the GSB signal at a time delay of 1.4 ps. The same approach was applied to all other kinetic traces.^36^ Since the positions and decay time scales of the ESA signals are consistent with vibrational relaxation processes, as observed for hydrogenases and other metal carbonyl compounds reported in previous pump–probe and 2D-IR studies,^28–34^ our data suggests that the observed features originate from a hot electronic ground state.

Taken together, these observations lead us to the following explanation of the initial events that take place following electronic excitation of the sample with the UV pump pulse:

(1) Assuming a high density of low-lying electronic states, excitation at 350 nm likely populates an initial excited state that rapidly decays through a sequence of lower-lying ones by ultrafast internal conversion.
(2) Consistent with this idea, most of the electronically excited molecules return rapidly and nonradiatively to the electronic ground state, leading to a hot ground state with vibrational energy distributed across a bath of vibrational modes. From there, the energy is dissipated directly toward the surrounding protein environment, leading to a repopulation of the overall ground state within less than 5 ps. In addition, a fraction of the energy is selectively directed into the CO stretching mode on the same time scale. This unusual, bond-specific energy redistribution gives rise to positive spectral features (ESA) that grow within a few picoseconds and decay with a typical vibrational lifetime of a few tens of picoseconds. We assume that the observation of these features is related to the relaxation of the CO stretch mode through a limited number of Fe-centered vibrational modes.^29,30,37^

(3) The remaining electronically excited molecules undergo the photoreaction. Based on the decay model described above, we assume that this process starts from an intermediary electronic state (rather than the initially excited one) that is probably localized at the Ni center and dissociative with regard to the Ni–CO coordinate.

While the mechanistic details are unclear, the overall process then leads to the formation of the Ni_a_-S state, which does not relax on the picosecond time scale. By evaluating the remaining intensity of the GSB signal relative to its initial amplitude, the quantum yield of the photoproduct is estimated at ca. 8%.

Formation of Ni_a_-S is evident from the long-lived component of the GSB peak of the Ni-SCO state as well as the positive feature reflecting the CO stretch mode of the Ni_a_-S state (vide supra). Compared to the stretch mode of the intrinsic Fe-bound CO ligand of Ni-SCO, the latter mode is slightly shifted toward higher frequencies, due to a decreased electron density at the active site, which results from the loss of the extrinsic Ni-bound CO ligand. Since the intense GSB band and the much weaker light-induced Ni_a_-S state band are only separated by 2 cm^–1^, it is difficult to extract an exact time constant for the photochemical formation of Ni_a_-S that is not affected by the GSB recovery. However, one can conclude that the Ni_a_-S state is formed within few tens of picoseconds (Figure 2), based on two arguments.

(1) Estimation of an apparent time constant leads to a value that is almost indistinguishable from that of the slower GSB recovery of the Ni-SCO parent state (Figure S3). If Ni_a_-S formation was significantly slower or faster, its time evolution should be separable, despite overlap with that GSB peak.
(2) No growth of the Ni_a_-S signal is observable after ca. 80–100 ps. Assuming that an exponential process can be considered completed after about four times the associated time constant, we conclude that Ni_a_-S is formed by CO photolysis from Ni-SCO within 20–25 ps or less. If the excited-state energy surface was dissociative in the Franck–Condon region, photolysis would be nearly instantaneous. Irrespective of the exact mechanism, the stretching vibration of the photolyzed CO cannot be observed, due to its expected high frequency and, in particular, small transition dipole moment.^38–41^ This observation is consistent with previous (steady-state) studies at cryogenic conditions, none of which reported an IR-spectroscopic signature of the photolyzed CO for hydrogenase.^6,24,42^

While picosecond time scales are dominated by photophysical relaxation and photo product formation, as detailed above, longer time scales reflect rebinding of the CO ligand to Ni_a_-S and recovery of Ni-SCO. No significant dynamics were detected on the nanosecond time scale (Figure S4), indicating slow rebinding of the CO ligand. From microsecond data, which were recorded for delays from 10 to 980 μs (Figure 3A,B), we notice a slow decay of the negative GSB peak and the positive signal reflecting the Ni_a_-S photo product. These observations reflect the recovery of the Ni-SCO state and the decay of the Ni_a_-S state, with associated time constants of ca. 0.6 and 0.5 ms, respectively (Figure 3C). The two time constants are indistinguishable within the accuracy of the experiment, indicating that Ni_a_-S is directly converted back to Ni-SCO by rebinding of the CO ligand from a nearby position inside the active-site protein cavity. In principle, the obtained time constants might be slightly affected by sample refreshment. However, in that case, the obtained values would represent a lower limit for the rebinding rate and the time window accessible for H_2_ binding after CO photolysis.

**Figure 3:**
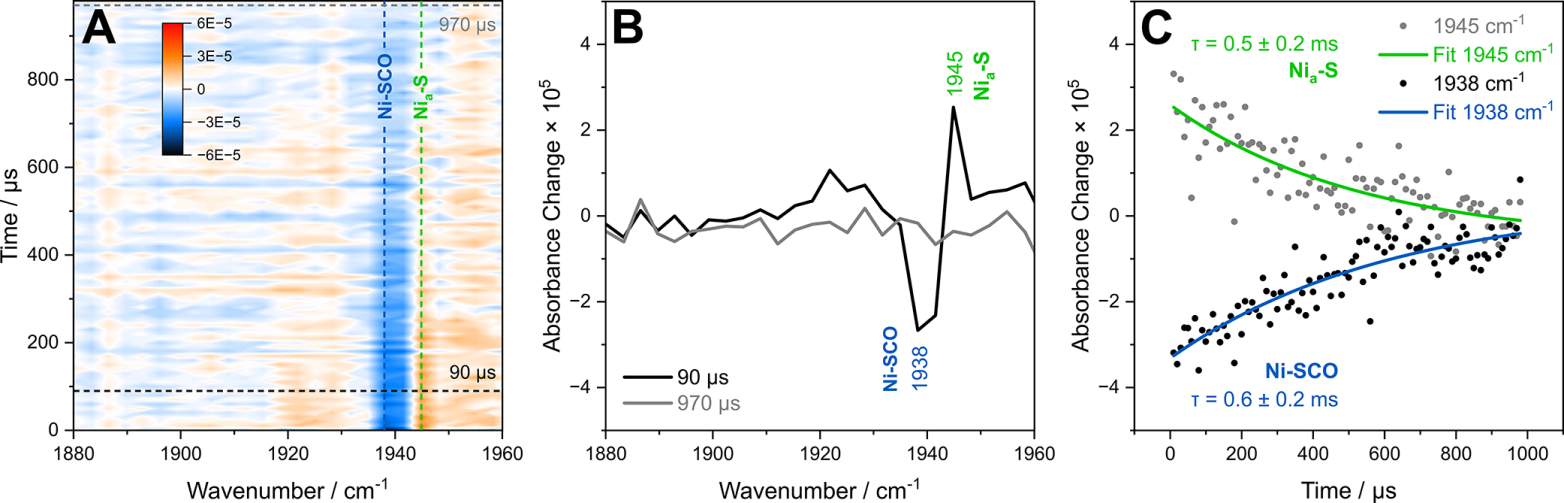
UV_pump_–IR_probe_ data of the [NiFe] hydrogenase from *Dv*MF, enriched in the Ni-SCO state, recorded over microsecond time scales after 350 nm excitation. (A) Contour plot of all time-resolved spectra. (B) Selected spectra obtained at *t* = 90 μs and *t* = 970 μs, respectively. (C) Time-evolution of signal intensities obtained at the indicated frequencies and corresponding fits.

In the present article, we have studied the dissociation and rebinding of an extrinsic CO ligand at the active site of the [NiFe] model hydrogenase from *Desulfovibrio vulgaris* Miyazaki F. While the CO-bound Ni-SCO state is catalytically inactive, photochemical CO photolysis yields the H_2_-binding Ni_a_-S state, which represents the starting point for H_2_ oxidation by [NiFe] hydrogenase. Our data demonstrate that reversible photochemical activation happens within 25 ps or less, and CO rebinding occurs on the order of 0.5 ms. These observations provide a time window of at least 8 orders of magnitude for studying H_2_ binding and cleavage after photochemical activation of the enzyme. Notably, this approach is reversible and not subject to intramolecular or intermolecular diffusion limitation. In addition, the rate of CO photolysis is on the order of the those predicted as a lower limit for the earliest steps of H_2_ binding^44^ and oxidation, and CO rebinding exceeds the turnover time of the enzyme.^45^ Thus, CO photolysis in the presence of H_2_ promises to allow probing the entire catalytic cycle, including the earliest and unexplored steps of H_2_ binding and activation.

The formation and stability of the CO-inhibited state, required for the introduced approach, may vary across [NiFe] hydrogenases. For instance, some O_2_-tolerant [NiFe] hydrogenases have been reported to be insensitive toward CO inhibition.^46,47^ However, this claim has been discussed controversially,^48,49^ and CO inhibition is generally recognized as a widespread feature among [NiFe] hydrogenases.^1^ Thus, the general strategy introduced here is widely applicable, and the feasibility has been demonstrated for one of the most prominent model hydrogenases. We therefore expect this approach to fill the final gaps in our understanding of biological H_2_ oxidation by [NiFe] hydrogenases.

## Supplementary Material



## Notes

Additional experimental details and supplementary figures (PDF)
