# Supplementary material for: From Carbon-Monoxide Inhibition to Light Activation: Probing [NiFe] Hydrogenase Dynamics by Multiscale Time-Resolved Infrared Spectroscopy
Source: Inorg Chem. 2026 Jun 18;65(26):14413–8. doi: 10.1021/acs.inorgchem.6c01321 (PMC13343517; doi:10.1021/acs.inorgchem.6c01321)
Supplement: Supplementary file 1 [file ic6c01321_si_001.pdf]

Supplementary Information

From Carbon-Monoxide Inhibition to Light Activation: Probing [NiFe] Hydrogenase Dynamics by Multiscale Time-Resolved Infrared Spectroscopy

Malin Khalil[1], Elizaveta Kobeleva[1], Cornelius C. M. Bernitzky[1], Partha Malakar[2], Sayantan Bhattacharya[2], Sagie Katz[3], Hiroaki Matsuura[4], Ingo Zebger[3], Gregory M. Greetham[2], Hideaki Ogata[4], James A. Birrell[5] and Marius Horch[1]*

[1] Freie Universität Berlin, Department of Physics, Ultrafast Dynamics in Catalysis, Arnimallee 14, 14195 Berlin, Germany. E-Mail: marius.horch@fu-berlin.de

[2] STFC Central Laser Facility, Research Complex at Harwell, Rutherford Appleton Laboratory, Harwell Campus, Didcot, OX11 0QX, UK.

[3] Technische Universität Berlin, Department of Chemistry, Spectroscopic Characterization of Metalloproteins, Straße des 17. Juni 135, 10623 Berlin, Germany.

[4] University of Hyogo, Graduate School of Life Science, Koto 3-2-1, Kamigori, Ako, 678-1297 Hyogo, Japan.

[5] University of Essex, School of Life Sciences, Wivenhoe Park, Colchester, CO4 3SQ, UK.

Contents

Experimental Details p. S2

Supplementary Figures S1–S4 p. S3

Supplementary References p. S5

Experimental Details

Cultivation of *Desulfovibrio vulgaris* Miyazaki F and purification of its [NiFe] hydrogenase was performed as described previously.[1, 2] For spectroscopic measurements, samples were buffered in 50 mM Tris-HCl, pH 8.3, and concentrated to ca. 0.9 mM. For generation of the Ni-SCO state, the enzyme was first reduced with H_2 (1 bar, 4.5 h) inside an anaerobic tent (COY laboratory products Inc.). Subsequently, the reduced enzyme was incubated with CO (1 bar, 30 min).[3] Samples were frozen in liquid N_2 until further usage.

Time-resolved UV_{pump}-IR_{probe} spectra were recorded in time-resolved multi-probe spectroscopy ($TR^{M}PS$) mode using the LIFEtime laser system of the STFC Central Laser Facility. A detailed description of the setup and the methodology can be found in references 4-6. Briefly, a 100 kHz ultrafast laser based on a custom dual Yb:KGW system (Pharos, Light Conversion) pumped pump and probe optical parametric amplifiers (OPA) for UV and MIR generation. These OPAs provided broadband MIR probe pulses (ca. 200 cm^{-1}) centered at 1925 and 2070 cm^{-1} (0.05 μJ, ca. 200 fs, 100 kHz) and 350 nm UV pump pulses (0.6 μJ, ca. 300 fs, 1 kHz). The polarization of pump and probe pulses was set to magic angle. The probe light was detected using two 128-pixel MCT detectors, yielding a resolution of 2–3 cm^{-1}. The sample holder was rastered to avoid photodamage by the UV pump pulse. Samples were kept under anaerobic conditions at all times using a gas-tight transmission cell with two CaF_2 windows separated by a 50 μm Teflon spacer (ca. 20 μL sample volume). Experiments were performed at ambient temperature (21 °C).

The spectra were frequency-calibrated using various known states of hydrogenases as a reference *via* polynomial fitting. Picosecond and microsecond timeframes were recorded together while nanosecond data were obtained separately. Accumulation times were 10 s per delay point for picosecond and microsecond timescales and 20 s for nanosecond timescales. All shown spectra were baseline corrected *via* spline functions in Origin.

Supplementary Figures

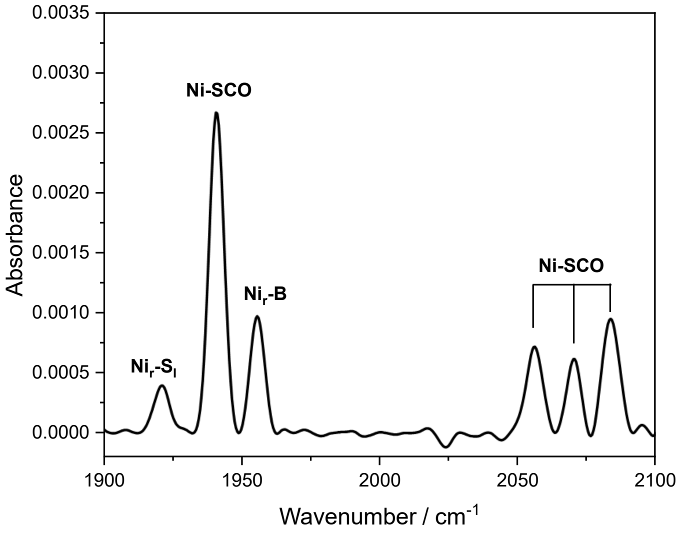

Figure S1. Representative FTIR spectrum of the [NiFe] hydrogenase from *Dv*MF enriched in Ni-SCO state. The spectrum was recorded from the sample as the TR^{M}PS data shown in the manuscript.

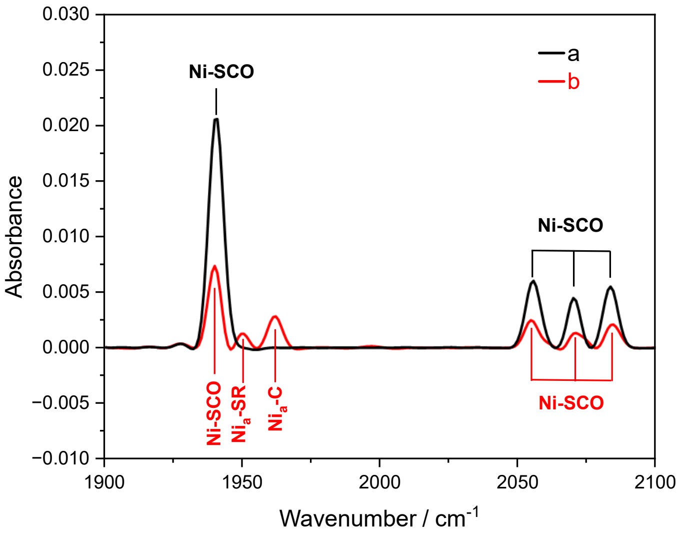

Figure S2. FTIR spectra of the [NiFe] hydrogenase from *Dv*MF enriched in Ni-SCO state (a) before incubation with H_2 and (b) after incubation with H_2. The spectra were recorded from a different sample than those shown in the rest of the manuscript. The preliminary data shown here indicate that ca. one third of the initially prepared Ni-SCO state remains stable in the presence of 100 % H_2. Notably, the procedure for maintaining the Ni-SCO state in the presence of H_2 has not been optimized yet. At such, we expect that a higher percentage can be retained under ideal conditions. In addition, we like to highlight that the absorbance of the remaining Ni-SCO population in the spectrum recorded *after* H_2 incubation is higher than that observed for the H_2-untreated sample that was used to record all other data (cf. Figure S1), demonstrating that high-quality data can be obtained from Ni-SCO in the presence of H_2.

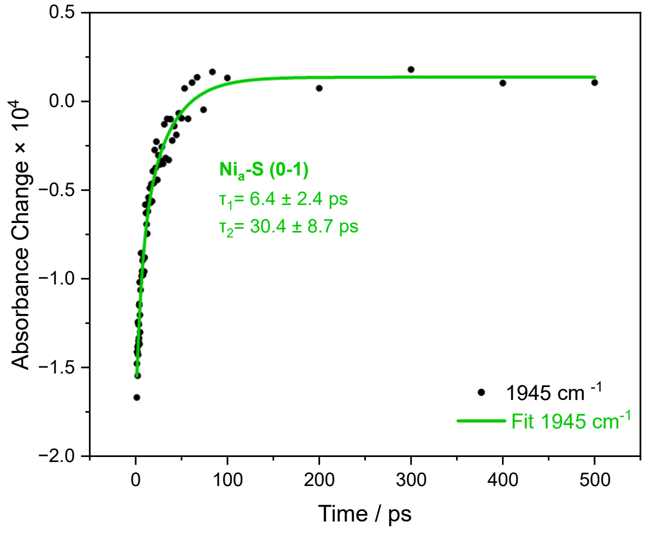

Figure S3. Time trace over picosecond timescales, obtained at the indicated frequency representing the Ni$_a$-S state.

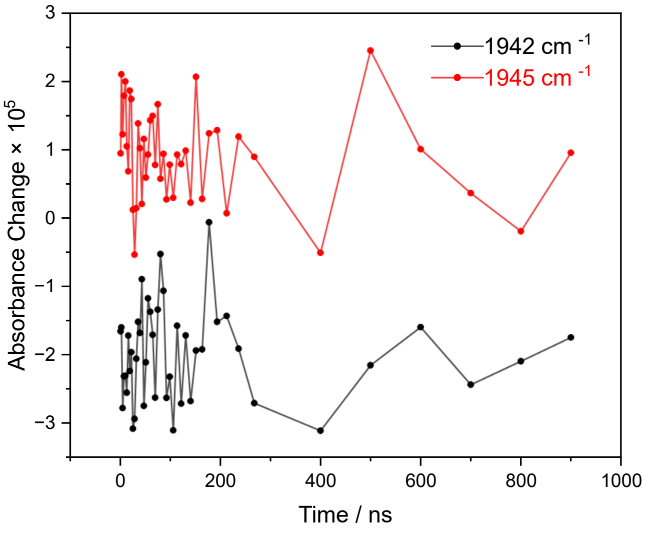

Figure S4. Time traces over nanosecond timescales, obtained at the indicated frequencies representing the parent state Ni-SCO (1942 cm^{-1}) and the product state Ni$_a$-S (1945 cm^{-1}) of the photolysis reaction.

Supplementary References

(1) Yagi, T.; Kimura, K.; Daidoji, H.; Sakai, F.; Tamura, S. Properties of Purified Hydrogenase from the Particulate Fraction of *Desulfovibrio vulgaris*, Miyazaki. *J. Biochem.* **1976**, *79* (3), 661-671.
(2) Higuchi, Y.; Yasuoka, N.; Kakudo, M.; Katsube, Y.; Yagi, T.; Inokuchi, H. Single Crystals of Hydrogenase from *Desulfovibrio vulgaris* Miyazaki F. *J. Biol. Chem.* **1987**, *262* (6), 2823-2825.
(3) Pandelia, M.-E.; Ogata, H.; Currell, L. J.; Flores, M.; Lubitz, W. Inhibition of the [NiFe] Hydrogenase from *Desulfovibrio vulgaris* Miyazaki F by Carbon Monoxide: An FTIR and EPR Spectroscopic Study. *Biochim. Biophys. - Bioenerg.* **2010**, *1797* (2), 304-313.
(4) Donaldson, P. M.; Greetham, G. M.; Middleton, C. T.; Luther, B. M.; Zanni, M. T.; Hamm, P.; Krummel, A. T. Breaking Barriers in Ultrafast Spectroscopy and Imaging Using 100 kHz Amplified Yb-Laser Systems. *Acc. Chem. Res.* **2023**, *56* (15), 2062-2071.
(5) Greetham, G. M.; Sole, D.; Clark, I. P.; Parker, A. W.; Pollard, M. R.; Towrie, M. Time-Resolved Multiple Probe Spectroscopy. *Rev. Sci. Instrum.* **2012**, *83*, 103107.
(6) Greetham, G. M.; Donaldson, P. M.; Nation, C.; Sazanovich, I. V.; Clark, I. P.; Shaw, D. J.; Parker, A. W.; Towrie, M. A 100 kHz Time-Resolved Multiple-Probe Femtosecond to Second Infrared Absorption Spectrometer. *Appl. Spectrosc.* **2016**, *70* (4), 645-653.
